# A novel monoclonal antibody of human stem cell factor inhibits umbilical cord blood stem cell ex vivo expansion

**DOI:** 10.1186/1756-8722-5-73

**Published:** 2012-12-10

**Authors:** Jie Fan, Xinxin Ding, Yongping Jiang

**Affiliations:** 1 Biopharmaceutical R&D Center, Chinese Academy of Medical Sciences & Peking Union Medical College, Suzhou 215126, China; 2 Biopharmagen Corp, Suzhou 215126, China

**Keywords:** Hematopoietic stem cell, Stem cell factor, Umbilical cord blood, Monoclonal antibody

## Abstract

Stem cell factor (SCF) activates hematopoietic stem cell (HSC) self-renewal and is being used to stimulate the ex vivo expansion of HSCs. The mechanism by which SCF supports expansion of HSCs remains poorly understood. In cord blood ex vivo expansion assays, a newly produced anti-SCF monoclonal antibody (clone 23C8) was found to significantly inhibit the expansion of CD34^+^ cells. This antibody appears to bind directly to a part of SCF that is critical for biological activity toward expansion of CD34^+^ cells, which is located in the first 104 amino acids from the NH_2_-terminus.

## Letter to the editor

The stem cell factor (SCF) is a principal cytokine involved in the expansion and differentiation of hematopoietic stem cells (HSCs) [1]. The combined use of SCF and other cytokines can effectively stimulate the ex vivo expansion of umbilical cord blood CD34^+^ cells [2-4]. The mechanisms of SCF’s action on HSCs are unknown. A better understanding of SCF’s action may help to further optimize systems for ex vivo expansion of HSCs.

Using a prokaryotic expression system, we obtained recombinant human SCF (rhSCF) for preparation of monoclonal antibodies (mAb) [5]. A specific mAb cell line (23C8) was identified, and utilized for studies on rhSCF. As shown in Table 1, rhSCF caused significant increases in CD34^+^ cell expansion index. When 23C8 was added together with rhSCF, the expansion index was reduced in an antibody concentration-dependent manner; at a molar ratio of mAb to rhSCF equal to or higher than 0.5, the activity of rhSCF was completely blocked. Thus, binding of 23C8 to rhSCF leads to inhibition of rhSCF’s biological activity.

**Table 1:** **Inhibitory effects of mAb 23C8 on CD34**^**+**^**cell ex vivo expansion**

**Additions**		**mAb:SCF molar ratio**	**MNC count at day 7 (×10**^**4**^**)**	**CD34**^**+**^**cells at day 7 (% total MNC)**	**CD34**^**+**^**expansion index**
**SCF**	**Anti-hrSCF**				
+ (reference)^*a*^	None	0	17.50 ± 1.50	31.44 ± 1.68	6.50 ± 0.87^#^
None^*b*^	None	0	5.00 ± 0.50	31.65 ± 0.50	1.87 ± 0.21*
+	Buffer only^*c*^	0	17.83 ± 0.58	29.12 ± 2.06	6.11 ± 0.41^#^
+	+	0.25	9.90 ± 0.93	31.64 ± 2.38	3.67 ± 0.09*^#^
+	+	0.50	5.00 ± 1.00	30.66 ± 0.39	1.80 ± 0.34*
+	+	1.0	4.50 ± 1.00	26.16 ± 1.27	1.39 ± 0.35*
+	+	2.0	4.50 ± 1.32	28.52 ± 4.41	1.52 ± 0.50*
+	+	5.0	4.67 ± 1.44	29.95 ± 4.00	1.60 ± 0.33*
+	+	10	4.25 ± 0.43	28.38 ± 1.71	1.43 ± 0.22*

Each well was added with umbilical cord blood MNC (~7.3 × 10^4^, containing 11.63% CD34^+^ cells), in 0.5 ml of STEM PRO®-34SFM medium containing 50 ng/ml rhTPO and 200 ng/ml rhFL on day 1. Additional reagents were added to various groups, as indicated, including 200 ng/ml reference rhSCF or rhSCF produced in-house, and mAb buffer alone or anti-rhSCF mAb added at different amounts. Cells were cultured at 37°C, with humidified air containing 5% CO_2_, for 7 days. Culture medium was replenished on day 3. Data represent means ± SD, n = 3. CD34^+^ expansion index is defined as the fold increase [(after expansion)/(before expansion)] in total CD34^+^ cell count.

^*a*^Positive control.

^*b*^Negative control.

^*c*^Vehicle control.

*, P < 0.05, compared with vehicle control and positive control.

^#^, P < 0.05, compared with negative control.

**Figure 1:**
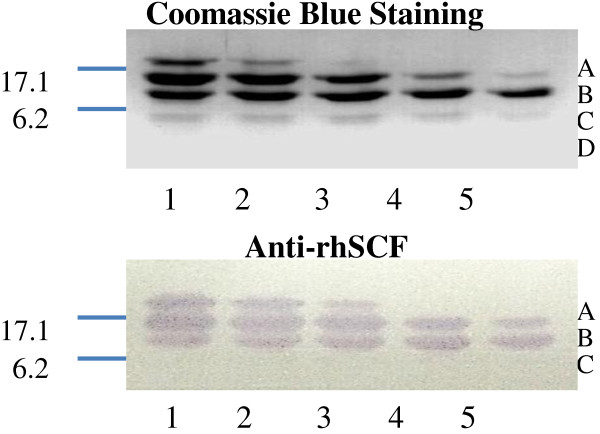
**Detection of tryptic peptides of rhSCF that contain the epitope recognized by the anti-rhSCF mAb.** Reaction mixtures of tryptic digest of rhSCF were analyzed on SDS-PAGE and stained with Coomassie Blue (*top*) to visualize total protein, or on a Western blot (*bottom*) using the anti-rhSCF mAb (clone 23C8). Lanes 1–5, purified rhSCF digested with trypsin for 0.5, 1, 2, 4, and 6 h, respectively. The intact protein (A) and three proteolytic products (C-D) are indicated; D was not recognized by the anti-rhSCF mAb.

Four bands (A-D) were detected when purified rhSCF was first subjected to limited proteolysis with trypsin and then analyzed by SDS-PAGE (Figure 1). Band A had the same size as intact rhSCF (19 kD), whereas bands B-D represented three tryptic fragments. Bands B (~15 kD) and C (~12 kD), as well as band A, were detected on Western blots using mAb 23C8, while band D (<6 kD) was not detected. Amino-terminal sequence analysis indicated that B and C both contained the amino terminus of rhSCF. A computer analysis of SCF protein sequence revealed putative trypsin cleavage sites between residues 128 and 129 (yielding a ~15-kD fragment, corresponding to B) and between 104 and 105 (yielding a ~12-kD fragment, corresponding to C). Therefore, we conclude that fragment C contained the amino acids 1-104 of SCF.

In summary, we show that mAb 23C8 can neutralize the activity of rhSCF, apparently through direct binding to a part of rhSCF located within the first 104 amino acids from the NH_2_-terminus. Our novel findings will be valuable for further mechanistic dissection and optimization of the ex vivo expansion of HSCs.

## Competing interests

The authors declare that they have no competing interests.

## Authors’ contributions

FJ, XD, and YJ participated in research design. FJ conducted experiments and performed data analysis. FJ, XD, and YJ wrote or contributed to the writing of the manuscript. All authors read and approved the final manuscript.

## Authors’ information

FJ was a graduate student at Peking Union Medical College, China; XD is an Adjunct Professor at Peking Union Medical College, China and Professor of SUNY at Albany, USA; YJ is a Professor at Peking Union Medical College, China.
